# Is human herpesvirus 8 infection more common in men than in women? an updated meta-analysis

**DOI:** 10.1186/s12879-024-09346-5

**Published:** 2024-04-29

**Authors:** Haibo Gong, Shuai Zhang, Jinfa Dou, Jing Chen

**Affiliations:** Department of Dermatology, Henan Provincial People’s Hospital; People’s Hospital of Zhengzhou University, No. 7 Weiwu Road, Zhengzhou, Zhengzhou, Henan 450003 China

**Keywords:** Kaposi's sarcoma, Human Herpesvirus 8, Seroprevalence

## Abstract

**Background:**

Clinically, most patients with Kaposi's sarcoma (KS) are male, and several direct and indirect mechanisms may underlie this increased susceptibility in men, Kaposi's sarcoma-associated herpesvirus (KSHV), also known as human herpesvirus 8 (HHV-8), is considered to be the primary etiological agent responsible for KS. Thus, we propose the hypothesis that men are more susceptible to HHV-8 infection, leading to a higher incidence of Kaposi's sarcoma among males. A meta-analysis was conducted to evaluate the association between gender and HHV-8 seropositivity in the general population.

**Methods:**

A comprehensive literature search was performed using 6 online databases: PubMed, EMBASE, Cochrane library, Web of Science, CNKI, and Wanfang. Studies published before March 15, 2023, were included.

**Results:**

In all, 33 articles including 41 studies were included in the meta-analysis. In the included adult population. men had a higher risk of HHV-8 infection than did women in adult populations from all over the world (odds ratio [OR]: 1.08, 95% confidence interval [CI]: 1.01–1.15), but no differences were found in child population from all over the world (OR: 0.90, 95% CI: 0.79–1.01). There was a significant difference in HHV-8 seroprevalence between men and women in sub-Saharan Africa (SSA) adult population (OR: 1.15, 95% CI: 1.05–1.26). However, no significant differences were observed in sub-Saharan Africa (SSA) child population (OR: 0.90, 95%CI 0.78–1.03). As for other continents, the results showed no significant difference, such as the Asian population (OR: 1.03, 95%CI: 0.92–1.16). or the European and American populations (OR 1.01, 95%CI 0.87–1.17).

**Conclusion:**

There was a slight gender disparity for HHV-8 infection in the adult population. Among the adult populations from SSA and globally, men were more likely to be infected with HHV-8 than were women. However, no statistical significance was observed in the child populations from SSA and globally. In the future, the inclusion of more standardized studies may strengthen the results of this study.

**Supplementary Information:**

The online version contains supplementary material available at 10.1186/s12879-024-09346-5.

## Background

Kaposi's sarcoma (KS) is a complex angioproliferative neoplasm that has attracted the attention of researchers and clinicians for decades [1, 2]. It primarily affects the skin of the extremities, face, trunk, external genitalia, and oropharyngeal mucosa. Lymph nodes and internal organs, most notably the respiratory and gastrointestinal tracts, are also often involved. It was first described and named by Moritz Kaposi, an Austro-Hungarian dermatologist, in 1872 [3]. It is unclear why patients with various types of KS are predominantly male. Several factors such as hormonal factors, inherent differences in the immune system, and high-risk behaviors may contribute to this phenomenon. Kaposi's sarcoma-associated herpes virus (KSHV), also known as human herpesvirus 8 (HHV-8) is considered a crucial factor in the pathogenesis of KS [4, 5]. Whether HHV-8 seroprevalence differs between men and women and thus explains the male predominance of KS, is yet to be determined.

HHV-8 is the primary cause of several malignancies, including KS, primary effusion lymphoma (PEL), and multicentric Castleman disease (MCD) [4]. Understanding the seroprevalence of HHV-8 is essential for assessing the burden of this virus and developing strategies to prevent the associated diseases.

Begré et al. [6] conducted a meta-analysis on this in 2016 and concluded that there was a slight gender disparity in the incidence of KS in sub-Saharan Africa (SSA). However, their findings may be outdated, since more relevant original articles on this issue have been published, we believe that the conclusion may be different now. In this meta-analysis, we not only included more English articles, but also included studies from the Asian continent in Chinese databases. Subgroup analyses were conducted separately in populations from different continents. Therefore, it is necessary to provide a more comprehensive evaluation of this issue. We conducted an updated meta-analysis to comprehensively evaluate the association between gender and HHV-8 seropositivity.

## Methods

### Search strategy

We searched the main English and Chinese language databases. Two of our researchers (Hai-bo Gong and Shuai Zhang) conducted a literature search of the PubMed, EMBASE, Cochrane library, Web of Science, CNKI, and Wanfang databases for articles published before March 15, 2023. The electronic search strategy of PubMed was as follows: ((((((((((((("Herpesvirus 8, Human"[Mesh]) OR HHV-8) OR KSHV) OR Kaposi's Sarcoma-Associated Herpesvirus) OR Kaposi's Sarcoma Associated Herpesvirus) OR Sarcoma-Associated Herpesvirus, Kaposi) OR Herpesvirus, Kaposi's Sarcoma-Associated) OR Herpesvirus, Kaposi's Sarcoma Associated) OR Human Herpesvirus 8) OR Herpesvirus, Kaposi's Sarcoma-Associated) OR Kaposi's Sarcoma-Associated Herpesviruses) OR Sarcoma-Associated Herpesviruses, Kaposi's)) AND ((((((seroprevalence) OR "Seroepidemiologic Studies"[Mesh])) OR ((Epidemiology) OR "Epidemiology"[Mesh])) OR ((incidence) OR "Incidence"[Mesh])) OR ((Prevalence) OR "Prevalence"[Mesh])).

### Inclusion and exclusion criteria

We collected data from cross-sectional studies on HHV-8 seroprevalence worldwide. The recruited participants in the included studies were representative of the general local population. The following information was extracted from the included studies: proportion of seropositive individuals by sex, race, and age. Therefore, studies conducted on blood donors, hospital-based studies, and studies conducted on specific populations, such as men who have sex with men, patients with solid organ transplants, HIV-positive individuals, those using intravenous (IV) drugs, or those who were incarcerated, were excluded.

### Data extraction

Two researchers, Gong Haibo and Zhang Shuai, independently extracted all the information from the included literature, including the authors, year of publication, country and region, total number of participants, number of men, number of women, frequency of seropositivity, frequency of seronegativity, age composition of the participants (children or adults), and number of seropositive individuals. Methods for the detection of HHV-8 and antibodies used in the detection process were also recorded. In case of disagreement, the two authors discussed the issue and submitted it to a third author for adjudication.

### Statistical analyses

This meta-analysis was conducted in accordance with Preferred Reporting Items for Systematic Review and Meta-Analyses (PRISMA) guidelines [7]. Both the Q-statistical test and I^2^ test were used to calculate between-study heterogeneity [8, 9]. In general, random- and fixed-effects models were used to combine the data in the presence (*p* < 0.1, I^2^ > 40%) or absence of heterogeneity (*p* > 0.1, I^2^ < 40%). The results of I^2^ statistic and Q statistic are often inconsistent in the actual calculation process. Because the number of studies in different subgroup analysis varies greatly, I^2^ statistic results will not change with the change of the number of studies, when in this situation, we used the I^2^ statistic to determine whether the heterogeneity was significant. Stata version 12.0 (StataCorp LP, College Station, TX, USA) was used to generate the forest and Egger’s plots.

## Results

### Number of eligible, included, and excluded studies

**Fig. 1:**
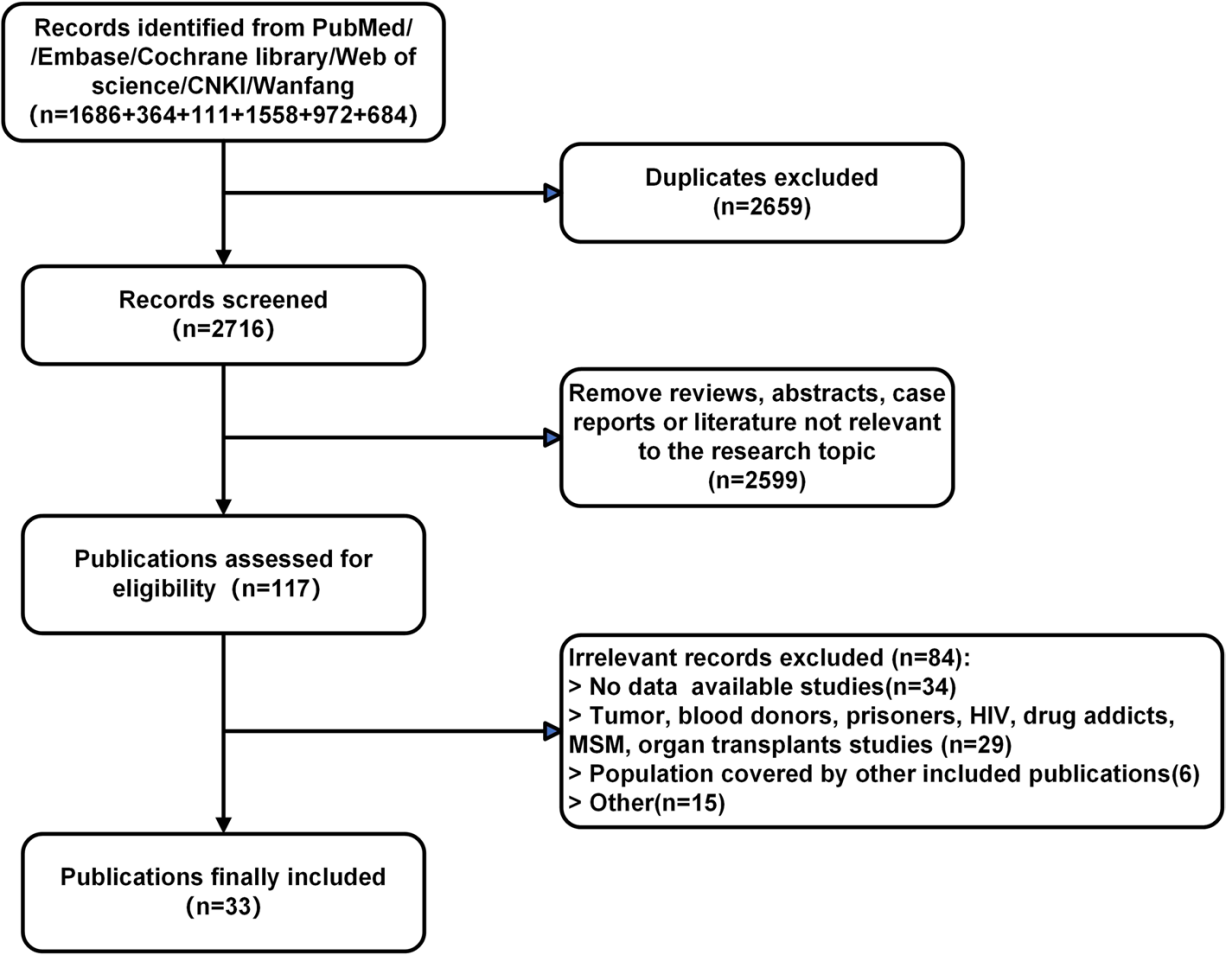
Flow diagram of literature search and screen

Using our search strategy, we searched six different databases, including the four main English language databases, PubMed, EMBASE, Cochrane and the Web of Science, and the two main Chinese language databases, CNKI and Wanfang. We then eliminated duplicate studies, and further eliminated abstracts, case reports, reviews, and other irrelevant studies that were not related to the content of this issue. Ultimately, 33 articles were included, of which 28 were in English and 5 in Chinese [10–42]. The literature identification process is illustrated in Fig. 1.

### Characteristics of included studies

Forty-one study groups from 33 articles included 25,902 male and 28,052 female participants. Detailed information about each study is presented in Table 1. The studies were from five continents: 15 (45%) from Asia, 12 (36%) from Africa, 2 (6%) from North America, 1 (3%) from South America, and 3 (9%) from the Europe. Of these, 10 studies included only children and 20 studies included only adults. The ages of the participants in the remaining 11 studies were unspecified.

**Table 1:** Main characteristics of included studies

Author	Year	Country	Age	HHV-8 tested used	Sample size	HHV-8( +)		HHV-8(-)			
						Male	Female		Male	Female	
Anderson	2008	USA	Children	EIA	4166	27	35	2026			2078
Antony	2021	Gabon	Adult	IFA	1020	229	143	403			245
Biryahwaho	2010	Uganda	Adult	EIA	2715	712	793	526			684
Butler (1)	2011	Uganda	Children	EIA	1382	189	183	504			506
Butler (2)	2011	Uganda	Adult	EIA	1477	298	294	396			489
Dedicoat	2004	South Africa	Children	EIA	2497	127	160	1136			1074
Engels	2007	USA	Adult	EIA	13,894	141	166	6159			7428
Fu	2009	China	Adult	EIA	2228	199	228	891			910
Fang Yuan	2022	China	unspecified	ELISA	678	48	60	219			351
Malope	2008	South Africa	Adult	EIA	1146	197	336	218			395
Mbulaiteye	2003	Tanzania	Children	EIA	361	125	122	88			102
Mbulaiteye	2008	Egypt	Adult	EIA	730	52	125	183			370
Angela Nalwoga	2020	Uganda	unspecified	ELISA	825	49	41	357			378
Perna (1)	2000	Italy	Children	IFA	319	9	11	159			140
Perna (2)	2000	Italy	Children	IFA	651	52	40	280			279
Plancoulaine (1)	2000	French Guiana	Children	IFA	656	23	25	329			279
Plancoulaine (2)	2000	French Guiana	Adult	IFA	681	58	71	251			301
Plancoulaine (1)	2004	Cameroon	Children	IFA	309	67	73	86			83
Plancoulaine (2)	2004	Cameroon	Adult	IFA	299	105	119	29			46
Serraino	2003	Italy	Adult	IFA	200	9	6	91			94
Tedeschi	2006	Sweden	Adult	IFA	516	39	36	218			223
Wang	2011	China	Adult	EIA	1008	111	122	386			389
Wawer	2001	Uganda	Adult	IFA	522	102	99	137			184
Wen	2021	China	unspecified	ELISA	1078	96	167	324			487
Zheng Jun (1)	2017	China	Adult	IFA	700	136	112	228			224
Zheng Jun (2)	2017	China	Adult	IFA	594	105	142	139			208
Zhang Tiejun	2017	China	Adult	IFA	1583	96	66	887			534
Cao Yifei	2014	China	Children	IFA	178	47	39	61			31
Angela Nalwoga (1)	2019	Uganda	unspecified	ELISA	1571	646	561	155			209
Angela Nalwoga (2)	2019	Uganda	unspecified	ELISA	1310	641	450	103			116
Kay L. Crabtree	2017	Zambia	Children	IFA	270	65	72	76			57
Ryoko Awazawa	2017	Japan	Adult	ELISA	1132	97	77	489			469
Yuan Huangbo	2018	China	Adult	IFA	594	105	142	139			208
Zhang xin	2022	China	Adult	IFA	721	68	106	208			339
Zhang ying (1)	2013	China	unspecified	ELISA	1008	22	21	529			436
Zhang ying (2)	2013	China	unspecified	ELISA	100	20	20	402			527
Zhang ying (3)	2013	China	unspecified	ELISA	882	28	21	472			361
Fang yuan	2017	China	unspecified	ELISA	1000	87	103	293			517
Fang qin	2006	China	unspecified	ELISA	560	18	11	299			232
Zhu ye	2010	China	unspecified	ELISA	1281	38	55	643			545
He miao	2014	China	Adult	IFA	171	58	29	42			42

*EIA* Enzyme immunoassay, *IFA* Immunofluorescence assay, *ELISA* Enzyme linked immunosorbent assay

### Meta-analysis results

**Fig. 2:**
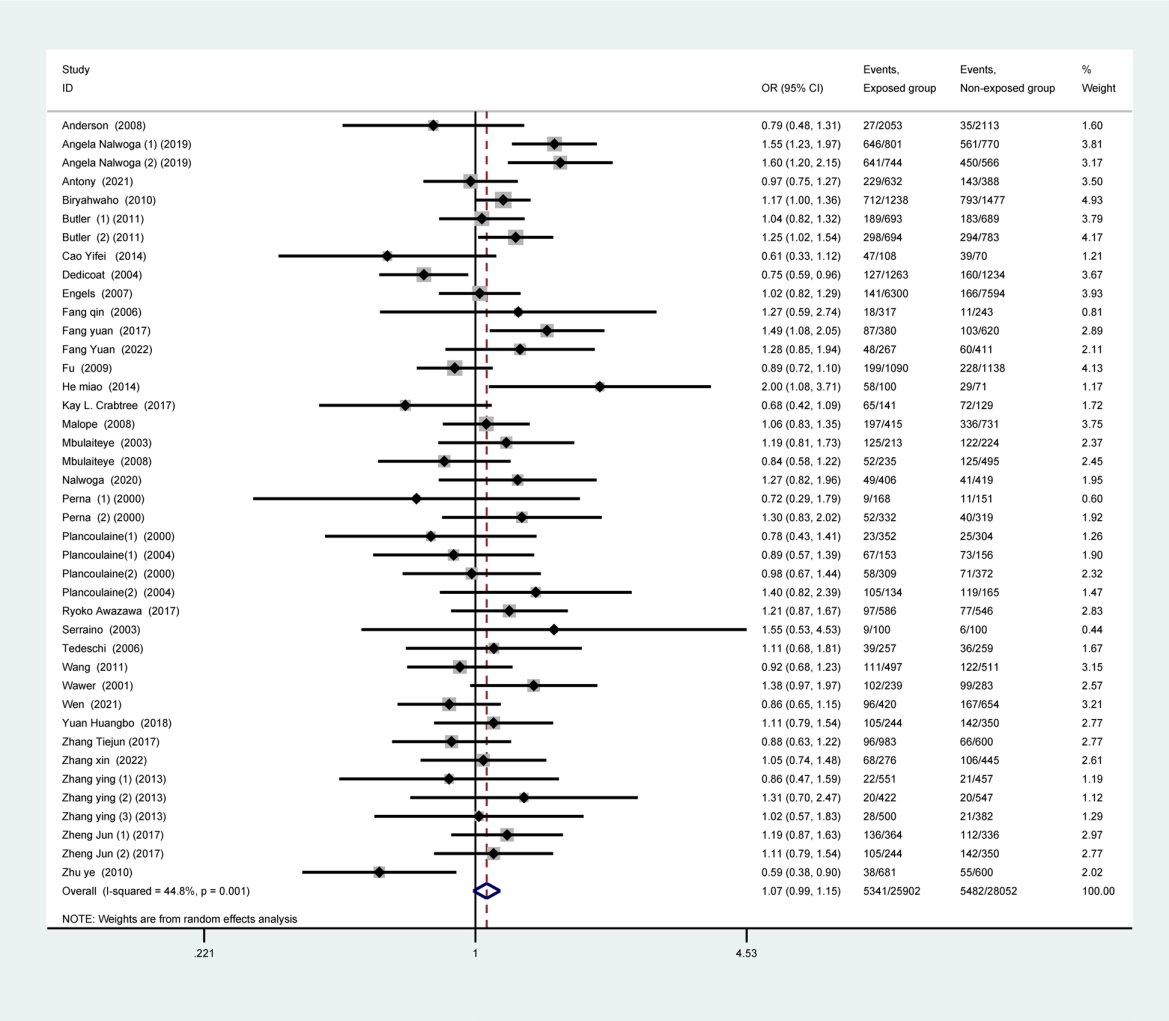
Forest plot of all included population worldwide

**Fig. 3:**
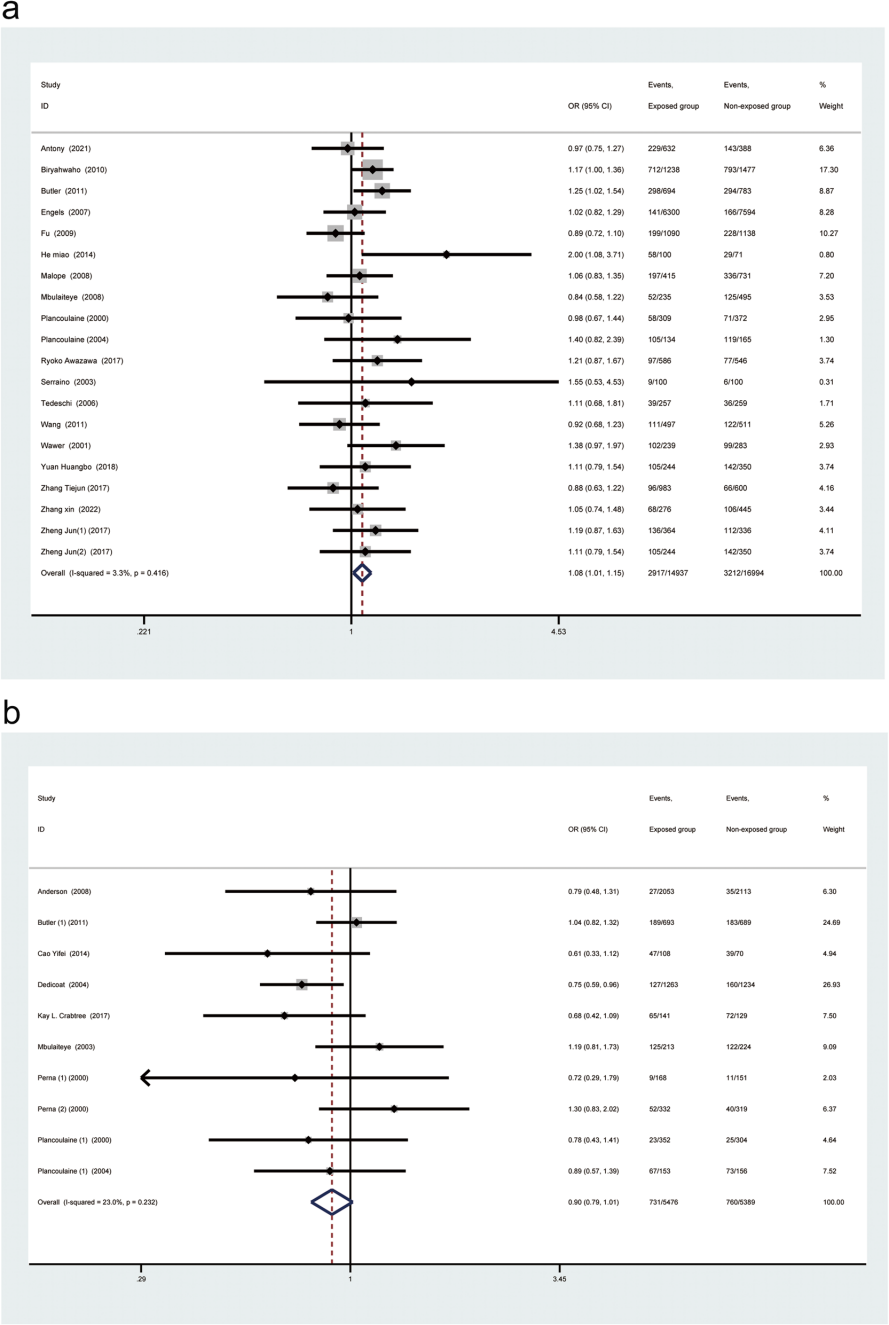
Subgroup analysis of all included studies worldwide stratified by age. a, Subgroup analysis of adult population included in all studies worldwide. b, Subgroup analysis of child population included in all studies worldwide

For the total included population from all over the world, random-effects models analyses showed no significant difference between gender and HHV-8 seropositivity (OR: 1.07, 95% CI: 0.99–1.15; Fig. 2), while the fixed-effects models showed that there was a significant association between gender and HHV-8 seropositivity (OR: 1.07, 95% CI: 1.02–1.13; Appendix.1). Because of some degree of heterogeneity between studies (I^2^ = 44.8%, *p* = 0.001), we finally selected the random-effects model results as the final calculation. For the total included adult population, there was a significant association between male gender and HHV-8 seropositivity (OR: 1.08, 95% CI: 1.01–1.15; Fig. 3a); however, no such association was found in the child population (OR: 0.90, 95% CI: 0.79–1.01; Fig. 3b).

**Fig. 4:**
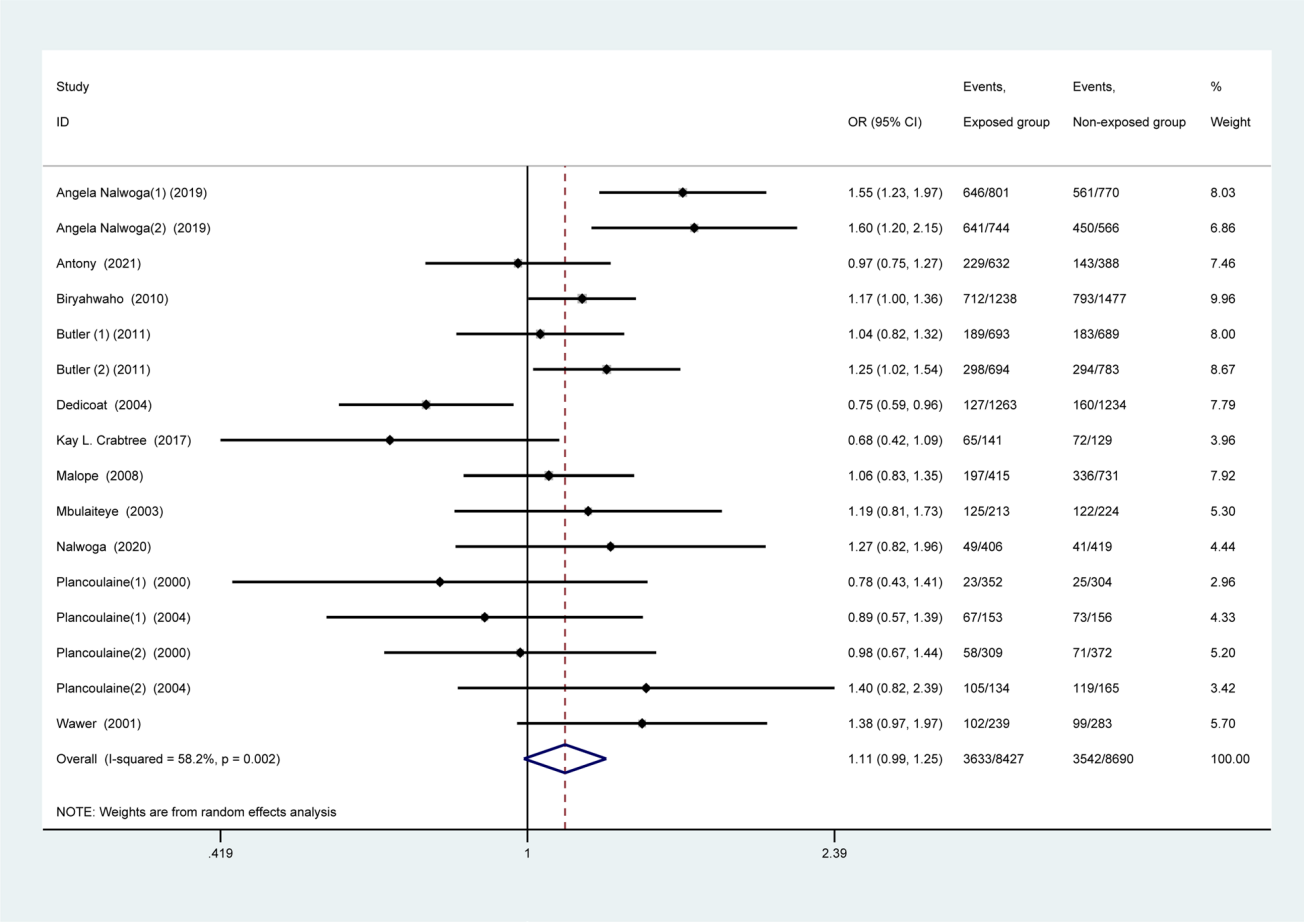
Subgroup analysis of population from Sub-Saharan Africa

**Fig. 5:**
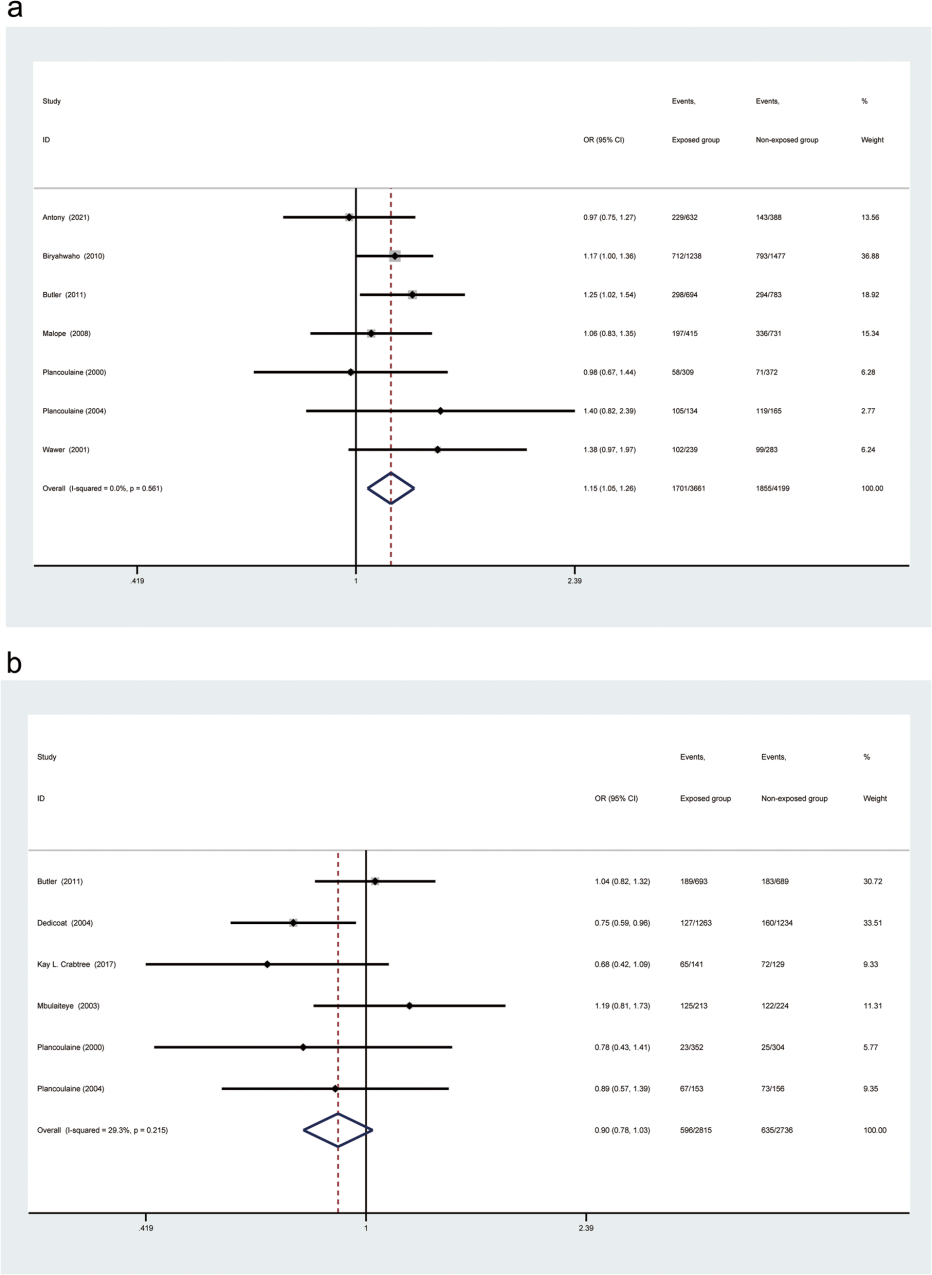
Subgroup analysis of populations from Sub-Saharan Africa stratified by age. **a**, Subgroup analysis of adult population included in studies from sub-Saharan Africa. **b**, Subgroup analysis of child population included in studies from sub-Saharan Africa

In the SSA region, For the total population (I^2^ = 58.2%, *p* = 0.002), the heterogeneity between studies was large enough so that we choose the random effect model for calculation. The results showed no significant difference (OR: 1.11, 95% CI: 0.99–1.25, Fig. 4). While using a fixed-effect model, it was found that male gender was associated with HHV-8 seropositivity in the total population (OR 1.13, 95% CI 1.05–1.21; Appendix.2). In the adult population subgroup (OR: 1.15, 95% CI: 1.05–1.26; Fig. 5a), but not in children (OR: 0.90, 95% CI: 0.78–1.03; Fig. 5b).

**Fig. 6:**
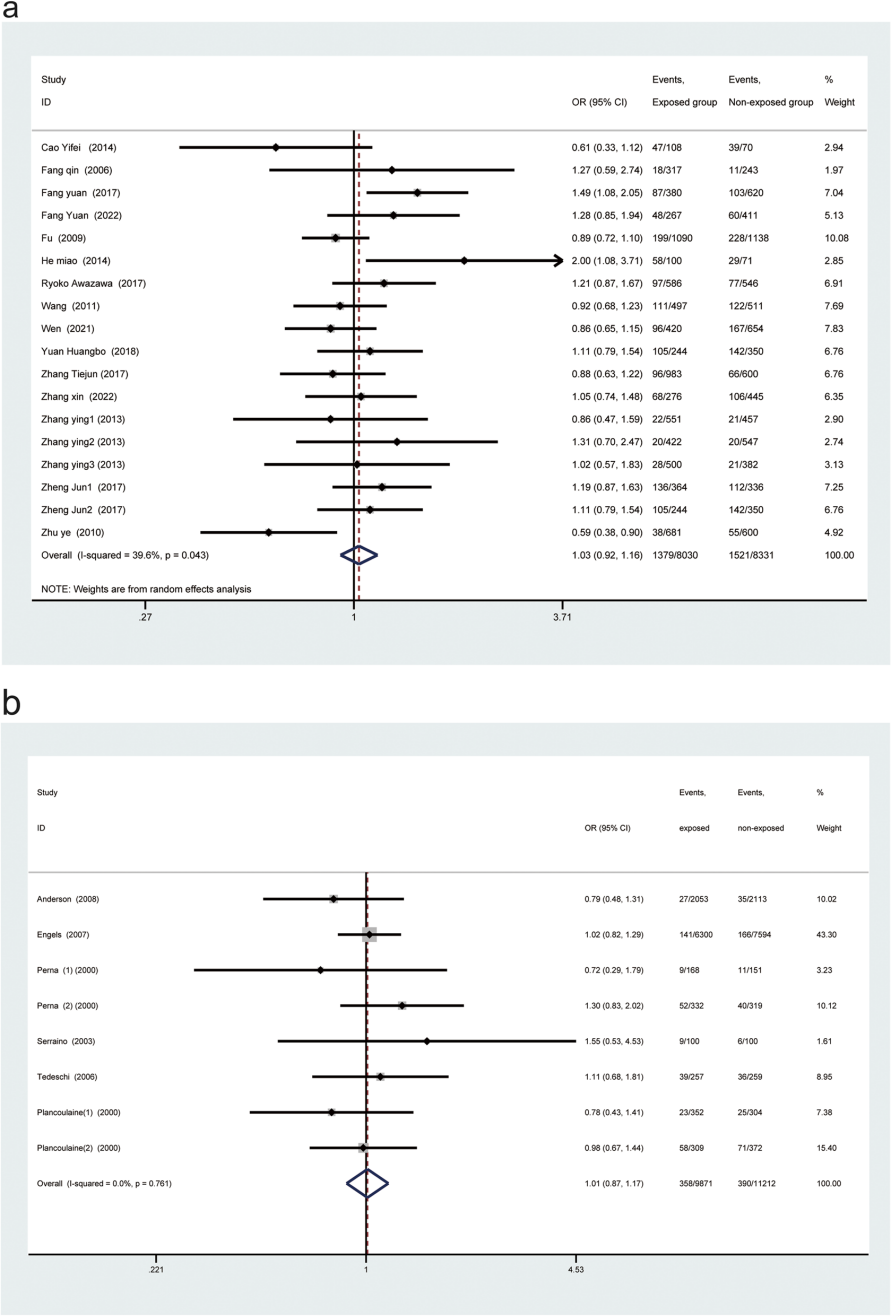
Subgroup analysis of populations from other continents. **a**, Subgroup analysis of population from Asia. **b**, Subgroup analysis of population from Europe and America

For other continents in the world, the results also showed that no statistically significant difference was observed (Asian region, OR: 1.03, 95% CI: 0.92–1.16; Fig. 6a; European and American population, OR: 1.01, 95%CI: 0.87–1.17; Fig. 6b).

### Sensitivity analysis and study bias

We removed the included studies individually to test the robustness and reliability of the results. The significance of the pooled ORs and 95% CIs did not change, indicating the stability of the results.

**Fig. 7:**
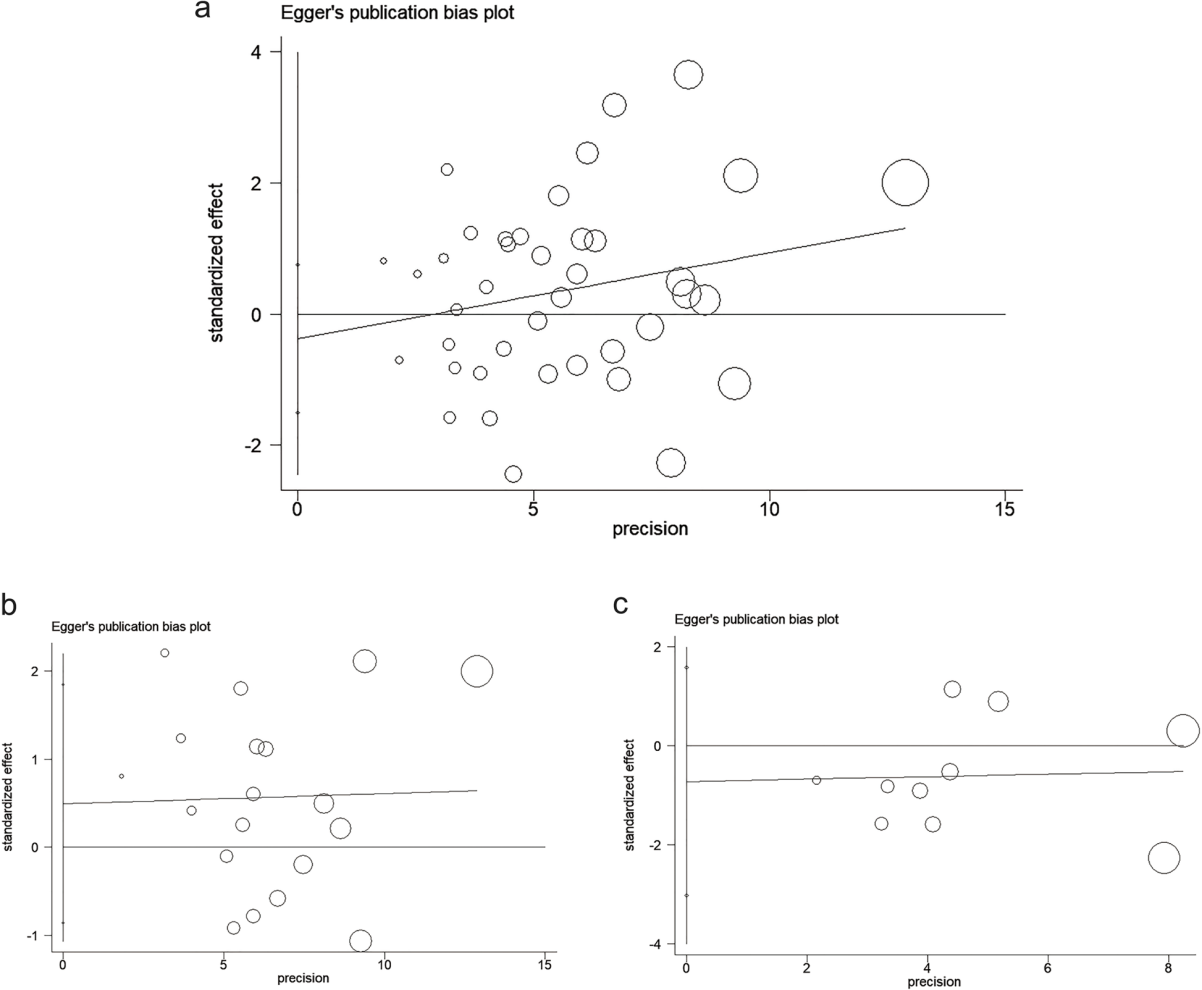
Publication bias for included studies worldwide. **a**, Publication bias for all included studies worldwide. **b**, Publication bias of adult population studies included worldwide. c, Publication bias of child population studies included worldwide

**Fig. 8:**
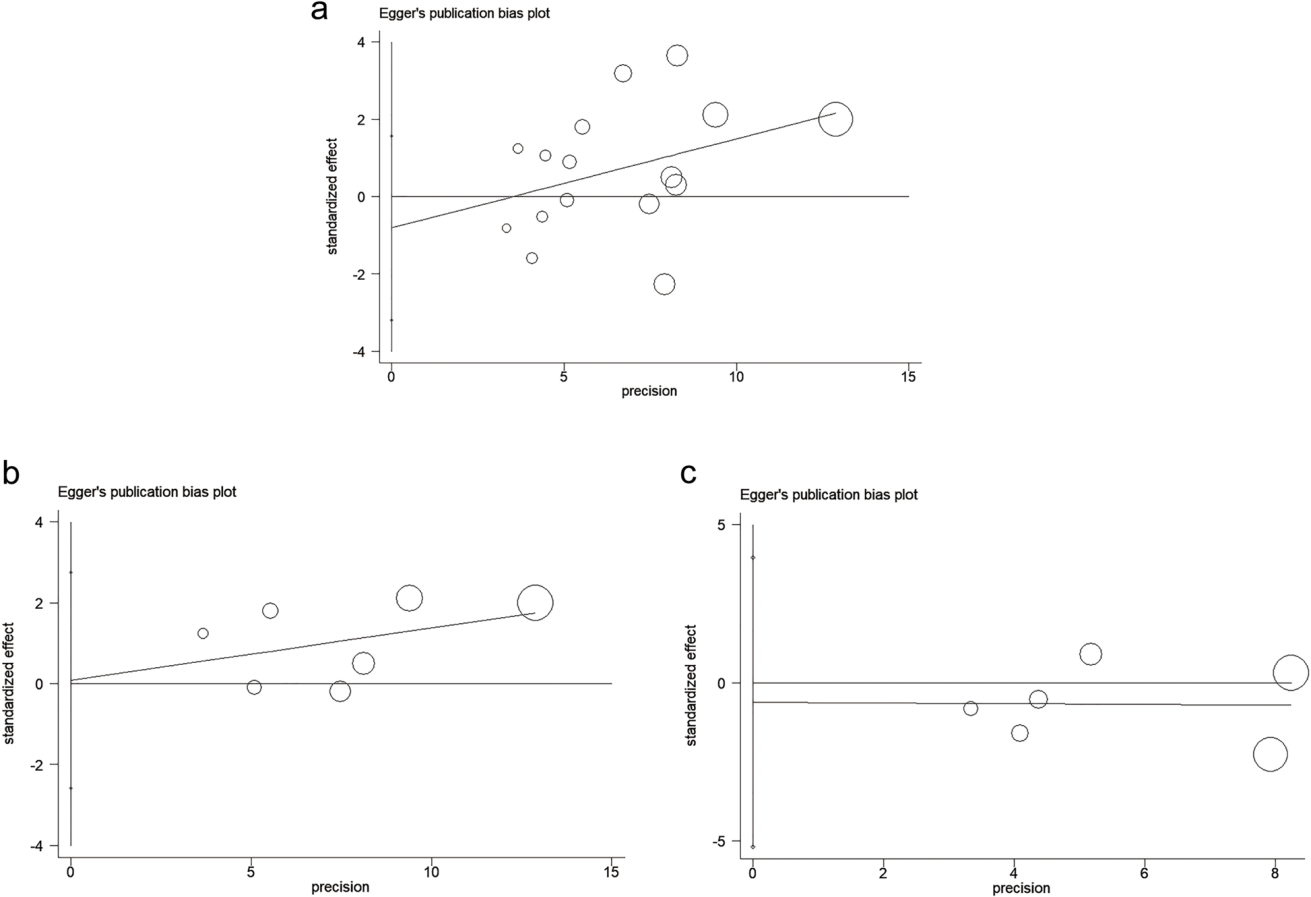
Publication bias for included studies in Sub-Saharan Africa. **a**, Publication bias for all included studies in SSA. **b**, Publication bias of adult population studies included in SSA. c. Publication bias of child population studies included in SSA

**Fig. 9:**
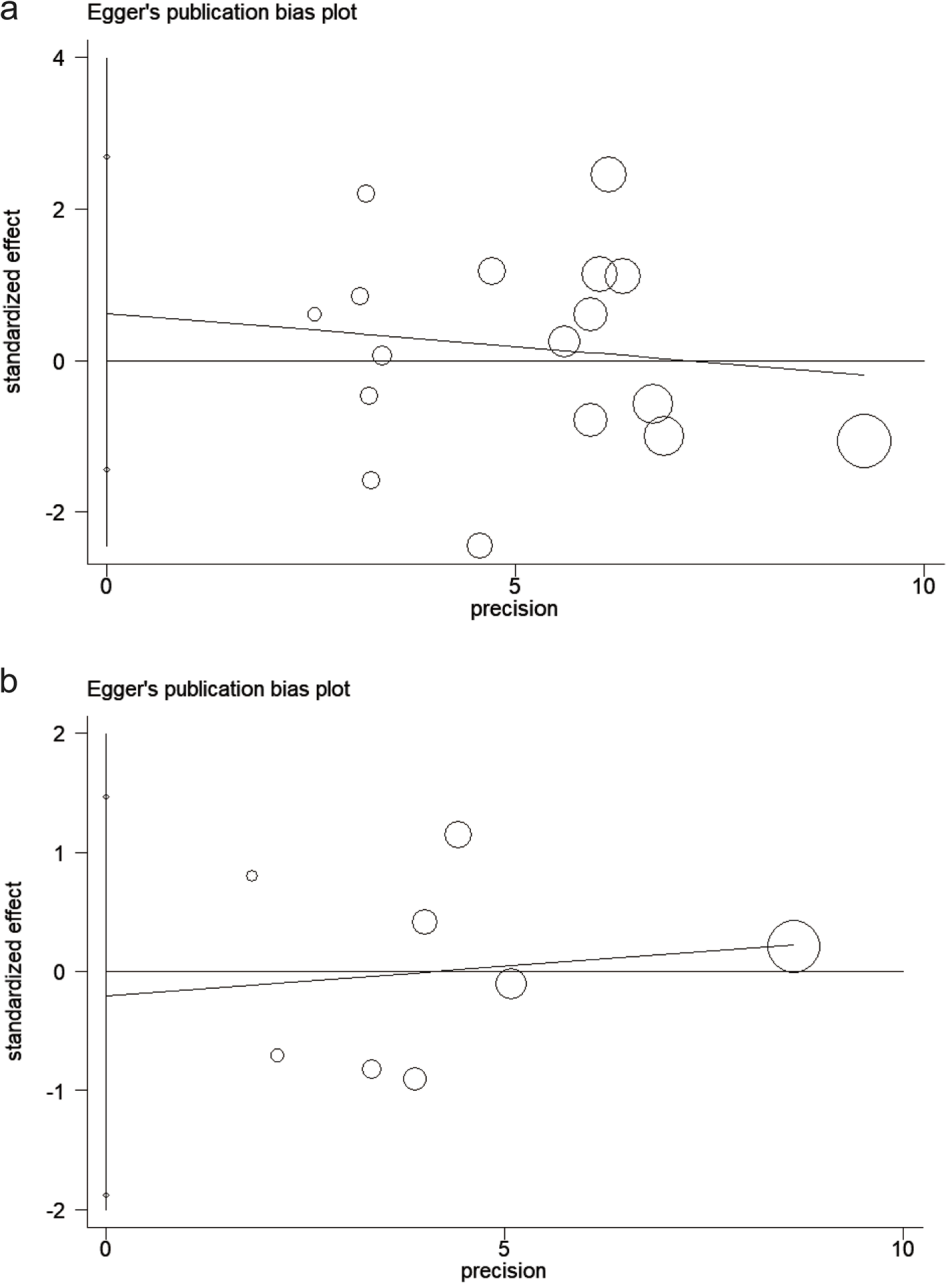
Publication bias for included studies in other continents. **a**, Publication bias for all included studies in Asia. **b**, Publication bias for all included studies in Europe and Americas

We used Egger's test to calculate the publication bias of the included studies in each subgroup analysis, and these results are shown in Figs. 7, 8, and 9. All the *p*-values of Egger’s test were > 0.1, indicating that there was no publication bias between the included studies in all group analyses. Including the total population from all over the world (*p* = 0.504, Fig. 7a); adult-only population from all over the world (*p* = 0.455, Fig. 7b); child-only population from all over the world (*p* = 0.489, Fig. 7c); total SSA population (*p* = 0.477, Fig. 8a); adult-only population from SSA (*p* = 0.939, Fig. 8b); child-only population from SSA (*p* = 0.730, Fig. 8c); Asian population (*p* = 0.531, Fig. 9a); and European and American population (*p* = 0.774, Fig. 9b).

## Discussion

This is an updated meta-analysis based on the work of Begré et al. [6]. To date, this meta-analysis is the most comprehensive. Our results suggested that HHV-8 infection is slightly more common in men than in women among the adult SSA population as well as the adult population from all over the world. However, in children across all populations, not just SSA, there were no results suggesting that boys were more likely to be infected with HHV-8 than girls. These results suggest that the male gender vulnerability of HHV-8 infection may not be related to genetic background, but to living habits and environmental factors in the region. The importance of the results from the fixed-effects model analysis of all included populations in SSA and all over the world should not be overlooked; we believe that when the quality of research is sufficiently standardized and the number of quantity researchers is large enough, we can apply the fixed-effects model and may conclude that there is a statistical difference.

The higher incidence of KS in men than in women may be attributed to a combination of immune system differences; hormonal, viral and genetic factors; and high-risk behaviors. Understanding these factors is crucial to developing better strategies for the prevention, early detection, and treatment of KS. The sero-epidemiologic distribution of HHV-8 may play a role in the pathogenesis of KS. It is probably not a coincidence that the results for the SSA adult population were consistent with those for all included adult population worldwide. However, this remains unclear because, clinically, far more male cases than female cases of KS have been encountered. Yet our results showed that men have only a weak predisposition to HHV-8 infection compared with women, since none of the ORs were very large. The statistical differences observed in our study were only slightly significant. Therefore, the higher number of men affected by KS compared to women is likely multifactorial, involving a combination of biological, behavioral, and social factors. The extent to which infection with the Kaposi's sarcoma-associated herpesvirus contributes to this phenomenon requires further research. There may be additional patterns underlying these results that remain to be understood.

This study has several limitations. Firstly, the male gender predominance in KS may have multifactorial causes. This study only examines this issue from the perspective of HHV-8 infection. Other contributing factors could include variances in immune system responses between men and women, hormonal influences, genetic predispositions, and gender-related behaviors. However, their exact impacts and roles remain unclear. Secondly, this study only included papers published in English and Chinese, excluding those published in other languages, which may introduce selection bias. Moreover, variations in technical methods, reagent manufacturers, age determination criteria for children in subgroup analyses, and the stringency of study population screening across different investigations could also introduce biases, thereby affecting the final outcomes. Thirdly, while we aimed to include all relevant studies on the global seroprevalence of HHV-8, the number of studies included was relatively limited. Further research with larger sample sizes and more comprehensive analyses is warranted.

## Conclusion

Adult populations from Sub-Saharan Africa (SSA), similar to adult populations worldwide, are more likely to test positive for HHV-8 seropositivity than women. However, no significant differences were observed among children from the same regions. These sero-epidemiological patterns of KSHV may help explain the higher prevalence of Kaposi's Sarcoma (KS) in men compared to women.

### Supplementary Information

**Supplementary Material 1.**

**Supplementary Material 2.**

## Data Availability

All data generated or analyzed during this study are included in this published article.

## Abbreviations

KS: Kaposi's sarcoma
PEL: Primary effusion lymphoma
SSA: Sub-Saharan African
