# Supplementary figures and images for: Is human herpesvirus 8 infection more common in men than in women? an updated meta-analysis
Source: BMC Infect Dis. 2024 Apr 29;24:454. doi: 10.1186/s12879-024-09346-5 (PMC11059601; doi:10.1186/s12879-024-09346-5)

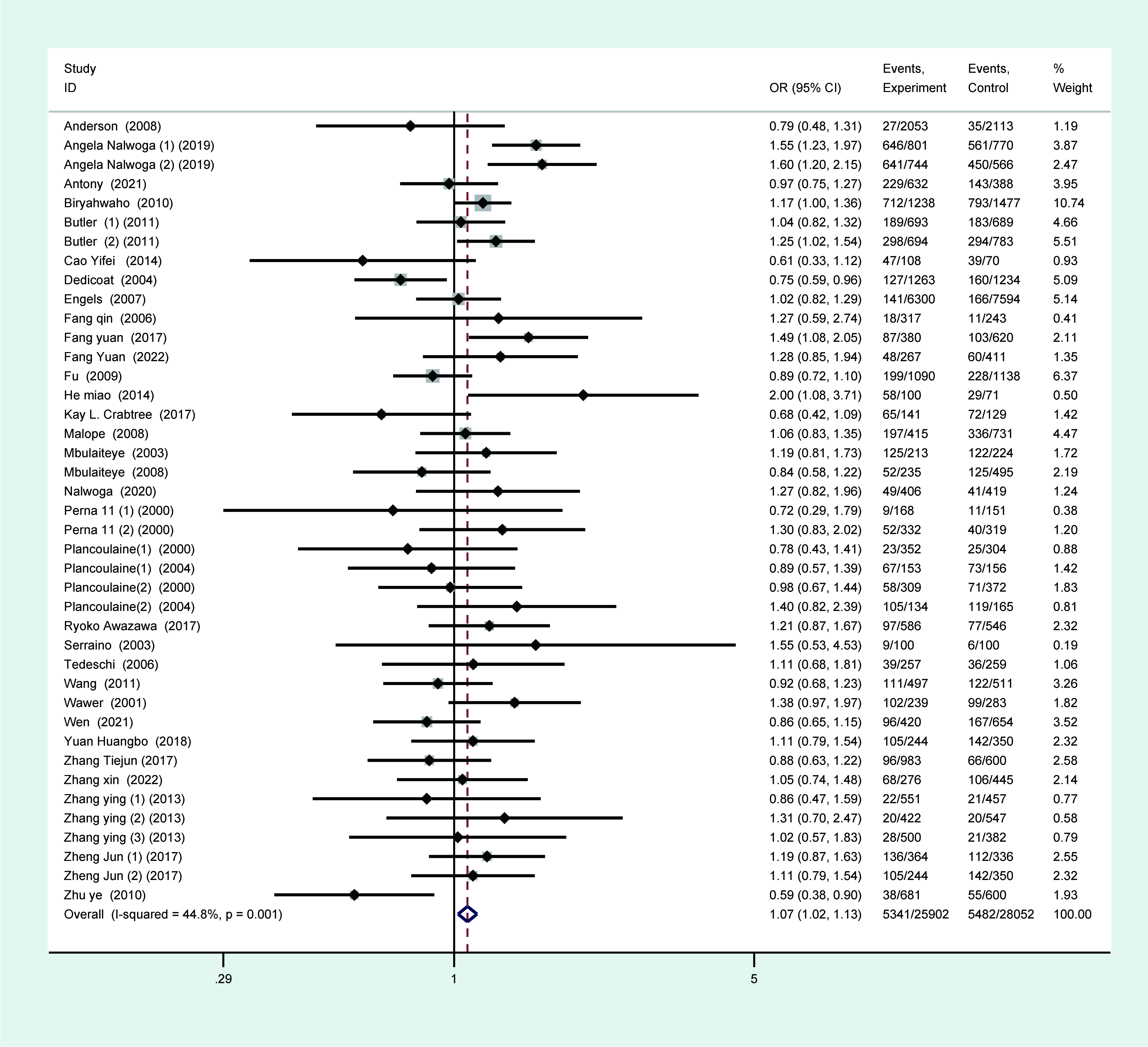

Supplement: Supplementary file 1 — Supplementary Material 1. [file 12879_2024_9346_MOESM1_ESM.tif]

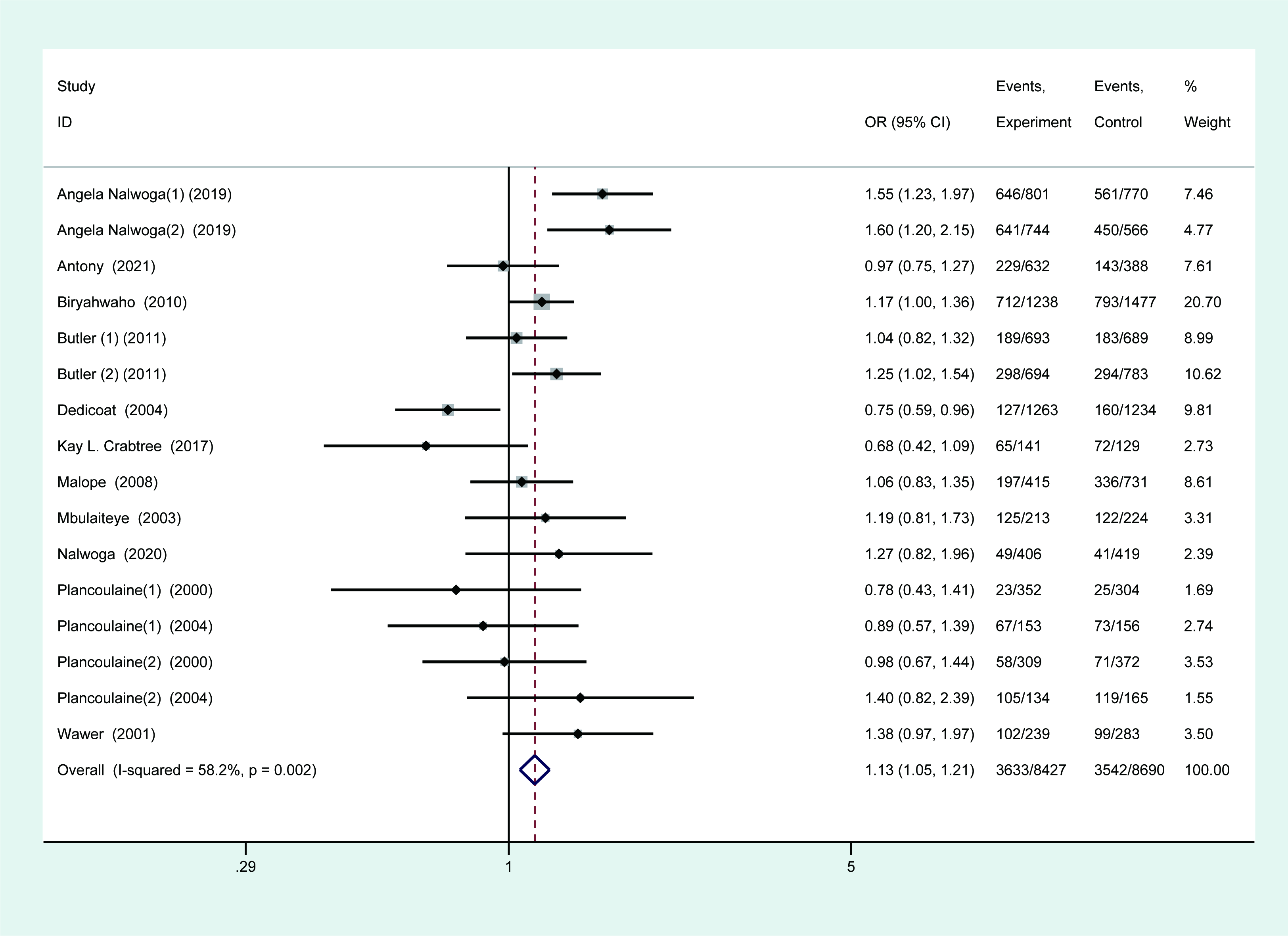

Supplement: Supplementary file 2 — Supplementary Material 2. [file 12879_2024_9346_MOESM2_ESM.tif]
